# Supplementary material for: Characterization of Potato Tuber Tissues Using Spatialized MRI T2 Relaxometry
Source: Biomolecules. 2023 Feb 2;13(2):286. doi: 10.3390/biom13020286 (PMC9953273; doi:10.3390/biom13020286)
Supplement: Supplementary file 1 [file biomolecules-13-00286-s001.zip › biomolecules-2172396-supplementary.pdf]

Supplementary Materials:

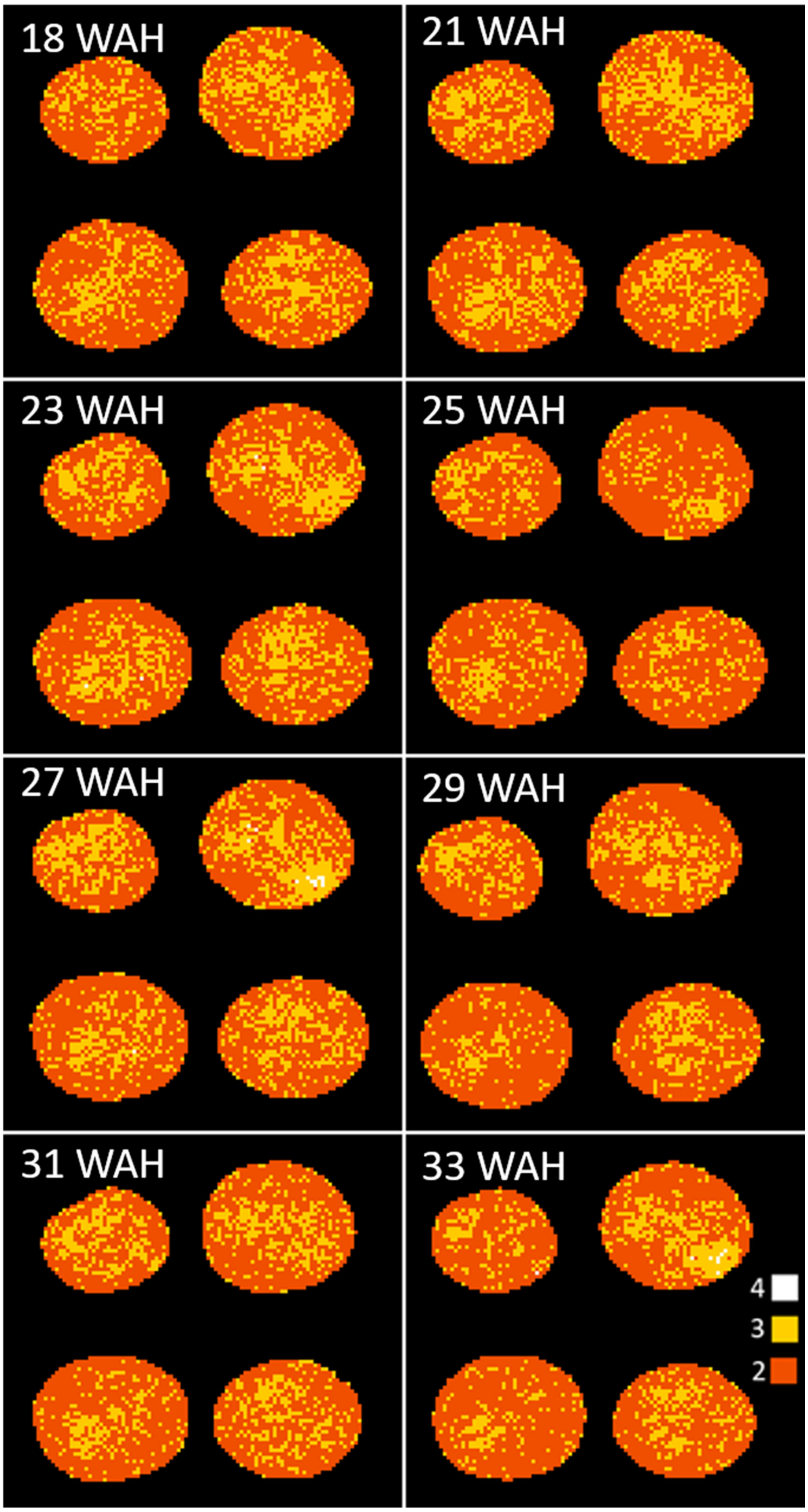

Figure S1. Optimal number, computed according to the Bayesian Information Criterion, of model components to estimate the relaxation parameters for each voxel for potato images recorded at 18, 21, 23, 25, 27, 31 and 33 WAH.

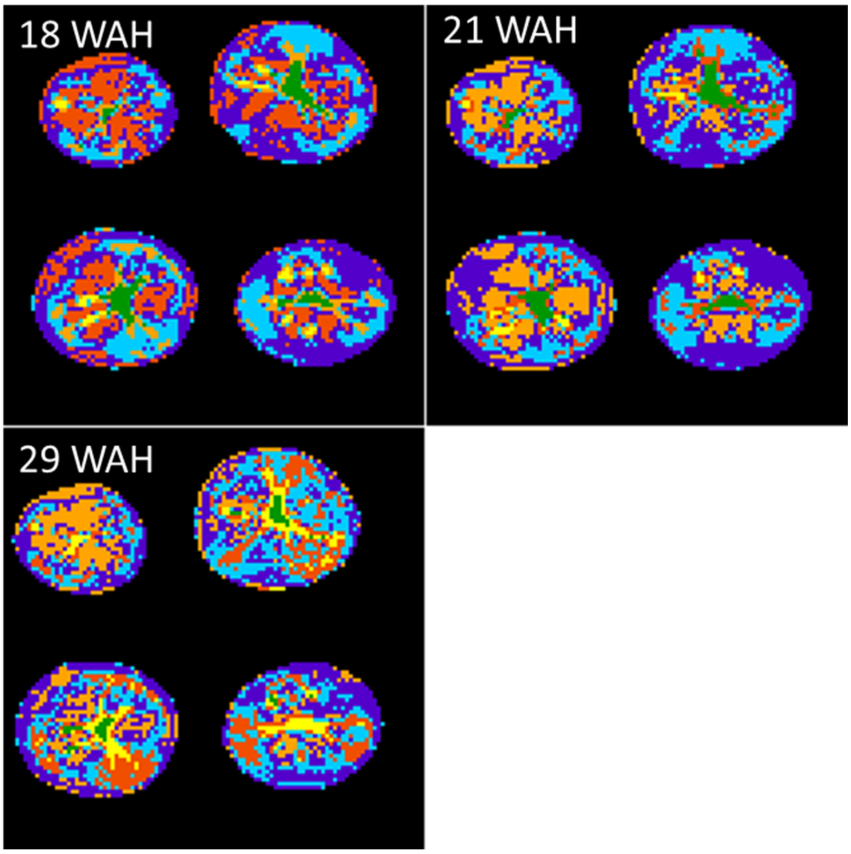

Figure S2. Classification results for 18, 21 and 29 WAH.

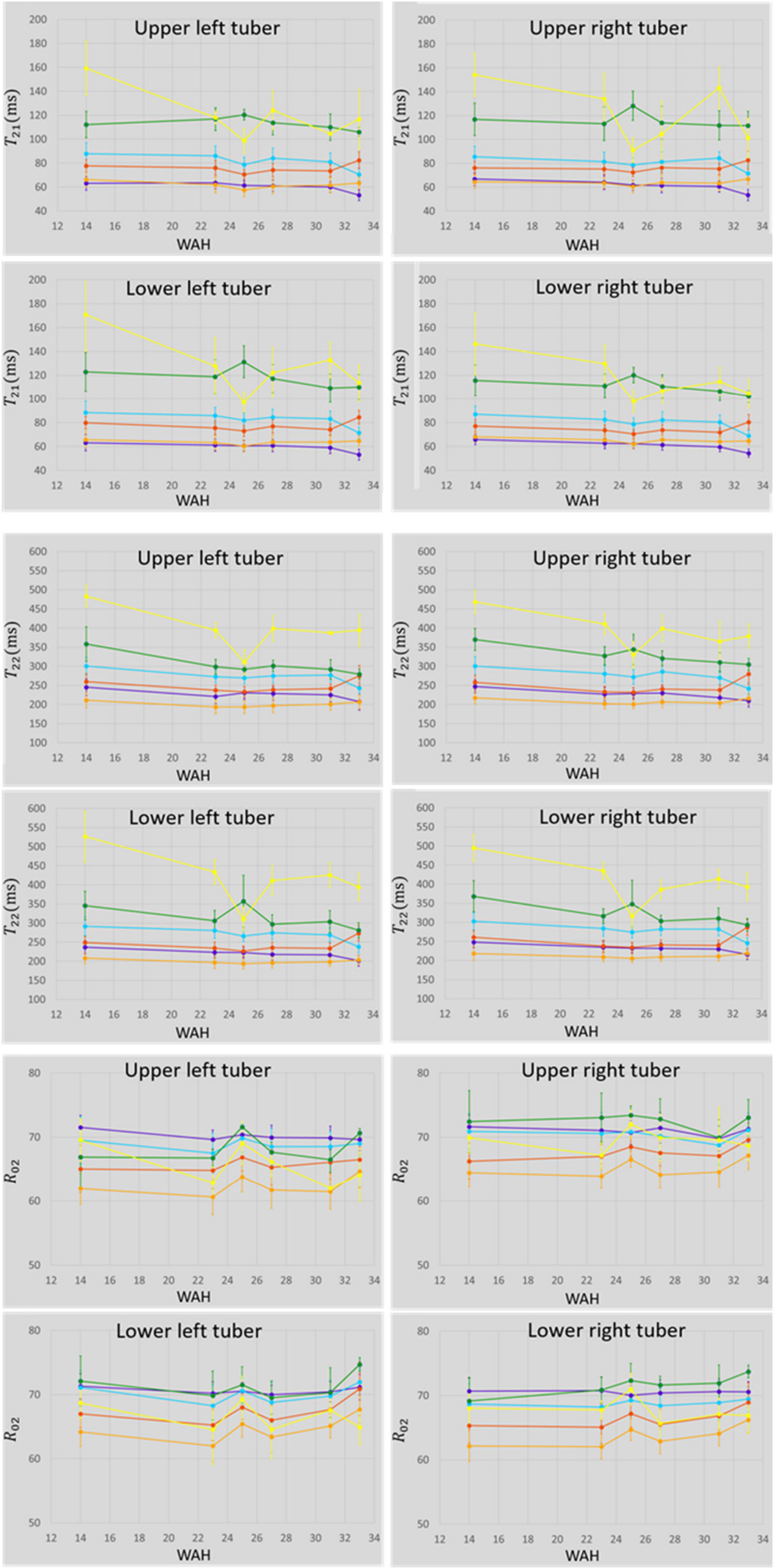

Figure S3. Evolution of the mean values of T_{21}, T_{22} and R_{02}, over time for each tuber individually. Error bars correspond to standard deviations. Colors were attributed regarding both T_{22} and spatial information as detailed in the text. On average, purple could be attributed to cortex, green to the pith and yellow to the defects, the three other colors, light and dark orange and cyan, to the flesh.
